# Müllerian duct anomalies with term pregnancy: a case report

**DOI:** 10.1186/s13256-020-02506-6

**Published:** 2020-11-02

**Authors:** Engku Ismail Engku-Husna, Nik Lah Nik-Ahmad-Zuky, Kadir Muhammad-Nashriq

**Affiliations:** Department of Obstetrics & Gynaecology, School of Medical Sciences, Universiti Sains Malaysia, Kubang Kerian, Kelantan 16150 Malaysia

**Keywords:** Müllerian duct anomaly, Congenital anomaly, Imperforated anus, Unicornuate uterus, Case report

## Abstract

**Background:**

Müllerian duct anomaly is a rare condition. Many cases remain unidentified, especially if asymptomatic. Thus, it is difficult to determine the actual incidence. Müllerian duct anomaly is associated with a wide range of gynecological and obstetric complications, namely infertility, endometriosis, urinary tract anomalies, and preterm delivery. Furthermore, congenital anomalies in pregnant mothers have a high risk of being genetically transmitted to their offspring.

**Case presentation:**

We report a case of a patient with unsuspected müllerian duct anomaly in a term pregnancy. A 33-year-old Malay woman with previously uninvestigated involuntary primary infertility for 4 years presented with acute right pyelonephritis in labor at 38 weeks of gestation. She has had multiple congenital anomalies since birth and had undergone numerous surgeries during childhood. Her range of congenital defects included hydrocephalus, for which she was put on a ventriculoperitoneal shunt; imperforated anus; and tracheoesophageal fistula with a history of multiples surgeries. In addition, she had a shorter right lower limb length with limping gait. Her physical examination revealed a transverse scar at the right hypochondrium and multiple scars at the posterior thoracic region, levels T10–T12. Abdominal palpation revealed a term size uterus that was deviated to the left, with a singleton fetus in a nonengaged cephalic presentation. The cervical os was closed, but stricture bands were present on the vagina from the upper third until the fornices posteriorly. She also had multiple rectal prolapses and strictures over the rectum due to previous anorectoplasty. An emergency cesarean delivery was performed in view of the history of anorectoplasty, vaginal stricture, and infertility. Intraoperative findings showed a left unicornuate uterus with a communicating right rudimentary horn.

**Conclusion:**

Most cases of müllerian duct anomaly remain undiagnosed due to the lack of clinical suspicion and the absence of pathognomonic clinical and radiological characteristics. Because it is associated with a wide range of gynecological and obstetric complications, it is vital for healthcare providers to be aware of its existence and the role of antenatal radiological investigations in its diagnosis. The presence of multiple congenital abnormalities and a history of infertility in a pregnant woman should warrant the exclusion of müllerian duct anomalies from the beginning. Early detection of müllerian duct anomalies can facilitate an appropriate delivery plan and improve the general obstetric outcome.

## Introduction

Müllerian duct abnormalities (MDAs) are a broad and complex spectrum of defects that are often associated with primary amenorrhea, infertility, endometriosis, and obstetric complications. Women with congenital uterine malformation usually experience a higher incidence of complications during pregnancy and delivery. An obstetrician should have a high index of suspicion for an undiagnosed congenital defect such as MDA in the presence of infertility with a background of maternal congenital malformations. Early diagnosis and recognition of the condition may allow proper planning of treatment to ensure a favorable obstetric outcome.

## Case presentation

We report a case of MDA diagnosed during an emergency cesarean section of a pregnant woman with multiple congenital anomalies. A 33-year-old Malay woman with 4 years of previously uninvestigated involuntary primary infertility had spontaneously conceived. She presented with acute right pyelonephritis in labor at 38 weeks of gestation. Of note, she has had multiple underlying congenital anomalies since birth and had undergone various surgeries during childhood. The abnormalities included hydrocephalus, for which she was put on ventriculoperitoneal shunting; an imperforated anus; and tracheoesophageal fistula with a history of multiples surgeries. In addition, she had a shorter right lower limb length with limping gait. A detailed ultrasound scan at 20 weeks of gestation revealed a grossly normal fetus. Her medication history was not significant, and her social and family history revealed no remarkable findings.

Clinically, she was alert and not in sepsis. Her vital signs were stable. However, she had a low-grade fever of 37.5 °C. She had a transverse scar on her right hypochondrium and multiple scars at the posterior thoracic region at levels T10–T12. Her abdominal examination revealed a term size uterus that was deviated to the left, with a singleton, nonengaged fetus in cephalic presentation. The result of a right renal punch was positive. Her cervix was unfavorable, with fibrotic stricture bands at the upper one-third of the vagina up to the fornices. She also had multiple rectal prolapses and strictures from a previous anorectoplasty. Her kidneys were of normal shape and size on her ultrasound examination. Her blood parameters, including her renal profile, were within normal ranges. She was administered a broad-spectrum intravenous antibiotic.

**Fig. 1:**
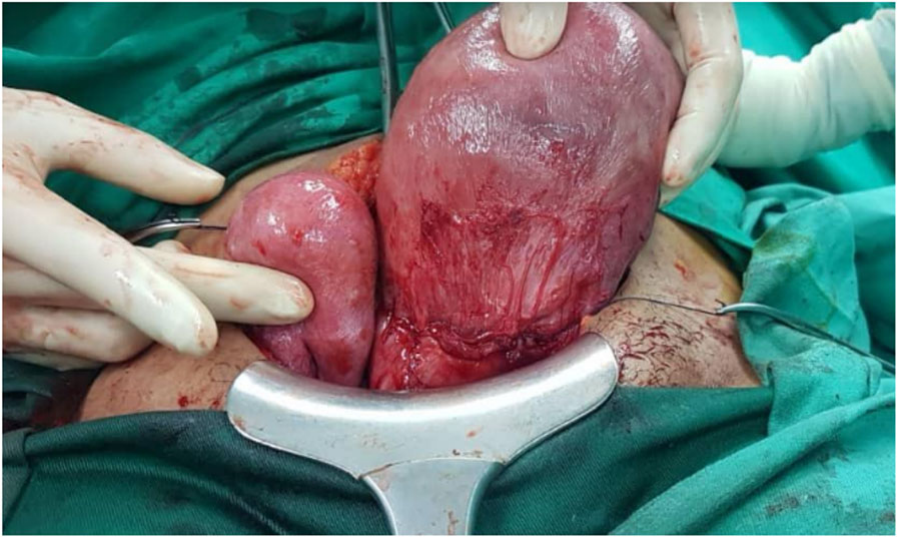
Intraoperative findings illustrating the left unicornuate uterus with a communicating right rudimentary horn (anterior view). The incision of the uterine segment was on the left side

**Fig. 2:**
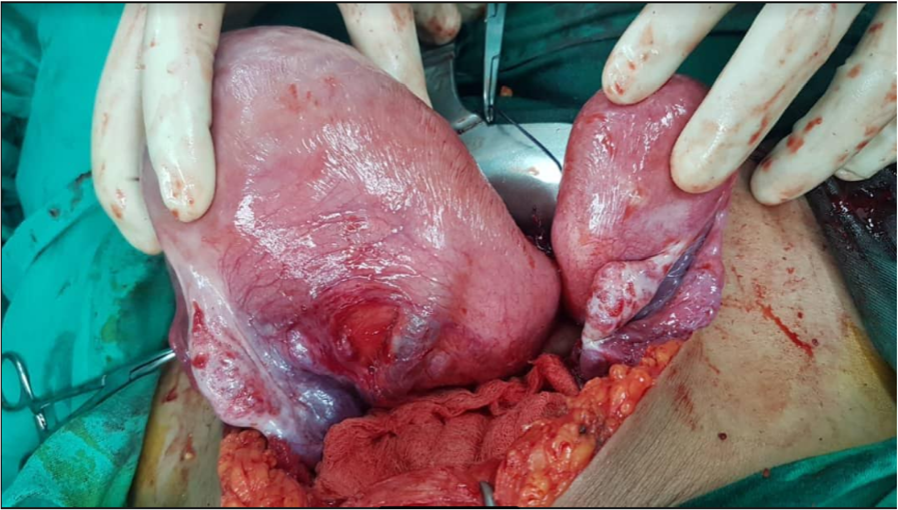
Intraoperative findings revealed a left unicornuate uterus with a communicating right rudimentary horn (posterior view). The patient’s fallopian tubes and ovaries were normal

In view of the above-mentioned maternal conditions, an emergency cesarean section was performed. Intraoperatively, MDA of left unicornuate uterus with fused right communicating horn was diagnosed. Both of the patient’s fallopian tubes and ovaries were normal (Figs. 1 and 2). A healthy and normal baby boy weighing 2500 g was delivered. He was vigorous at birth. Postoperatively, the intravenous antibiotic was continued for 3 days. Her condition improved, and the results of all cultures were negative. She was discharged on day 4 postoperatively and remained well at her 6-month follow-up.

## Discussion

The müllerian ducts are embryologic structures that exist in pairs before undergoing a mechanism of fusion and resorption *in utero* to form the uterus, fallopian tubes, cervix, and upper two-thirds of the vagina. MDAs are rare conditions. The actual prevalence of MDAs is difficult to ascertain because many of the cases are asymptomatic and thus remain undiagnosed. The reported prevalence of MDAs varies widely across studies. In a systematic review, the prevalence of MDAs was 5.5% in an unselected population, 8% in infertile women, 12.3% in women with a history of miscarriage, and 24.5% in women with a history of miscarriage and infertility [1].

The American Society for Reproductive Medicine (formerly the American Fertility Society) classification system for müllerian defects has been applied as the standard definition in the United States for decades [2]. When MDAs are diagnosed, any associated anomalies of the vagina, cervix, fallopian tubes, and renal system should also be identified and documented, even though they are not listed as part of the classification system. In a literature review regarding infertile and fertile women with MDAs, the frequencies of specific anomalies were septated uterus (35%t), bicornuate uterus (26%), arcuate uterus (18%), unicornuate uterus (10%), didelphys (8%), and agenesis (3%) [3].

MDAs are frequently associated with adverse gynecologic and obstetric complications, such as infertility, endometriosis, and miscarriage. They are also commonly associated with renal anomalies in as many as 30–50% of cases. The common renal anomalies are renal agenesis, ectopic kidney, hypoplasticity, fusion, duplication, and malrotation of the kidneys [4, 5]. Thus, the identification of both kidneys during the investigation is essential. MDAs are also frequently seen in women born with complex anorectal malformations. Other congenital abnormalities commonly associated with MDAs include vertebral body anomalies (29%) (that is, fused or wedged vertebral bodies, spina bifida; 22–23%), heart abnormalities (14.5%), and syndromes such as the Klippel-Feil syndrome (7%) [6]. Other conditions involving vertebral defects, anal atresia, cardiac defects, tracheoesophageal fistula, renal anomalies, and limb abnormalities (VACTERL association), such as renal or cardiac anomalies in patients with MDAs, could also affect their conception, fertility, modes of delivery, and obstetrical outcomes [7].

The diagnosis of MDAs remains challenging in most cases. Although hysterosalpingogram is useful in diagnosing a unicornuate uterus, it does not help in detecting a noncommunicating horn. Two-dimensional (2D) ultrasound is the initial imaging modality of choice because it is widely available, noninvasive, relatively inexpensive, and able to provide information about other relevant nonuterine structures such as ovaries, kidneys, and pelvic mass. It can also provide information on the subsequent imaging modality or modalities that should be chosen for definitive diagnosis. In contrast, three-dimensional (3D) reconstructed images provide more detailed information and often prevent the need for additional imaging [8, 9]. Historically, magnetic resonance imaging has been the gold standard for the diagnosis of reproductive tract anomalies [10]. However, magnetic resonance imaging is reserved for cases only when 2D or 3D ultrasound findings are limited and a definitive diagnosis is required to make decisions regarding patient care. Additional investigations include an intravenous pyelogram or renal ultrasonography to detect any horseshoe kidney, ipsilateral renal agenesis, or pelvic kidney. Laparoscopy is rarely indicated in the investigation of MDAs.

Among the various types of MDAs, the unicornuate uterus is associated with the poorest fetal survival. Even if the pregnancy is healthy, the obstetric performance is universally poor in this group. Common obstetrical complications include malpresentation, intrauterine growth retardation, and preterm birth. These reproductive problems are attributed to abnormal uterine vasculature and diminished myometrial mass of the unicornuate uterus [11]. As a result, cesarean delivery rates are high among this group of patients.

In view of these complications, it is crucial for female patients with multiple congenital anomalies to receive an early referral to exclude MDAs. A thorough assessment is compulsory during the initial pregnancy checkup to assess the severity of the condition. Early recognition and categorization of the condition allow appropriate management during the antepartum, intrapartum, and postpartum periods to ensure a good outcome for mother–baby dyad care.

However, our patient, even though she underwent regular antenatal review at the local health center, was referred only much later to our tertiary-level center for further evaluation. Her old admission records regarding her childhood conditions were not available. On the basis of limited records, her clinical findings were suggestive of vertebral, anorectal, tracheoesophageal, renal, and limb associations. Hence, the possibility of an underlying MDA should be highly suspected from the beginning. The MDA was proved intraoperatively with the presence of unicornuate uterus with a rudimentary horn and communicating uterine horn. This condition is frequently associated with ipsilateral renal and ureter agenesis [12]. However, it was absent in our patient.

## Conclusion

The majority of MDAs remain undiagnosed due to a lack of clinical suspicion and the absence of pathognomonic clinical and radiological characteristics. Hence, knowledge of its existence and the role of antenatal radiological investigations is vital to prevent the wide range of gynecological and obstetrical complications. As illustrated in our patient’s case, the presence of multiple congenital abnormalities and a history of infertility in a pregnant woman should warrant the exclusion of MDAs from the beginning. Early detection of MDAs will facilitate the most appropriate delivery plan to ensure the best maternal and neonatal outcomes.

## Data Availability

The patient’s information and medical records used for the case report are available from the corresponding author upon request.

## Abbreviation

MDA: Müllerian duct anomaly
